# Optimization of Maleinized Linseed Oil Loading as a Biobased Compatibilizer in Poly(Butylene Succinate) Composites with Almond Shell Flour

**DOI:** 10.3390/ma12050685

**Published:** 2019-02-26

**Authors:** Patricia Liminana, David Garcia-Sanoguera, Luis Quiles-Carrillo, Rafael Balart, Nestor Montanes

**Affiliations:** Technological Institute of Materials (ITM), Universitat Politècnica de València (UPV), Plaza Ferrándiz y Carbonell 1, 03801 Alcoy, Spain; patligre@mcm.upv.es (P.L.); dagarsa@dimm.upv.es (D.G.-S.); rbalart@mcm.upv.es (R.B.); nesmonmu@upvnet.upv.es (N.M.)

**Keywords:** polymer‒matrix composites (PMCs), mechanical properties, thermomechanical, electron microscopy, compatibilizers

## Abstract

Green composites of poly(butylene succinate) (PBS) were manufactured with almond shell flour (ASF) by reactive compatibilization with maleinized linseed oil *MLO) by extrusion and subsequent injection molding. ASF was kept constant at 30 wt %, while the effect of different MLO loading on mechanical, thermal, thermomechanical, and morphology properties was studied. Uncompatibilized PBS/ASF composites show a remarkable decrease in mechanical properties due to the nonexistent polymer‒filler interaction, as evidenced by field emission scanning electron microscopy (FESEM). MLO provides a plasticization effect on PBS/ASF composites but, in addition, acts as a compatibilizer agent since the maleic anhydride groups contained in MLO are likely to react with hydroxyl groups in both PBS end chains and ASF particles. This compatibilizing effect is observed by FESEM with a reduction of the gap between the filler particles and the surrounding PBS matrix. In addition, the T_g_ of PBS increases from −28 °C to −12 °C with an MLO content of 10 wt %, thus indicating compatibilization. MLO has been validated as an environmentally friendly additive to PBS/ASF composites to give materials with high environmental efficiency.

## 1. Introduction

Nowadays, there is growing interest in the development of biopolymers that could substitute for, or at least compete with, conventional petroleum-based polymers. This need is much more pronounced in the packaging industry due to the high volume of waste it generates. Among different alternatives such as proteins, polysaccharides, bacterial polymers, and so on, biopolyesters (either from petroleum origin or bio-derived) are gaining relevance as they are biodegradable (disintegrable in controlled compost soil conditions). One of these polyesters is poly(butylene succinate) (PBS), which presents interesting possibilities for manufacturing wood plastic composites (WPCs) or natural fiber-reinforced plastics (NFRPs) [1]. In general, the biodegradable synthetic polymers are mainly aliphatic polyesters produced by microbiological and chemical synthesis, natural polymer-based products, and their blends such as poly(lactic acid) (PLA), poly(ε-caprolactone) (PCL), poly(glycolic acid) (PGA), poly(butylene succinate) (PBS), poly(butylene succinate-co-adipate), among others. PBS offers interesting possibilities in the packaging industry due to an excellent combination of flexibility and biodegradability (more correctly, disintegration in controlled compost soil). PBS is produced through the condensation reaction of glycols such as 1,4-butanediol and succinic acid. Currently, PBS is obtained from the petroleum-based route, which involves the use of both 1,4-butanediol and succinic acid from petroleum sources. Nevertheless, it has been proposed as a renewable resource. It is worth remarking that some time ago the U.S. Department of Energy (DoE) classified succinic acid as one of the 12 most promising biobased building blocks for a biorefinery concept. Succinic acid can be obtained from renewable feedstocks such as glucose, sucrose, and glycerol. The main advantage is a lower carbon footprint. New fermentation processes are being developed in order to obtain a cost-effective material [2,3,4]. With regard to 1,4-butanediol, it can be obtained from the fermentation of dextrose, for example, and biobased BDO represents a valuable building block for a wide variety of engineering materials such as polyurethanes and biobased polymers, with interesting applications in the automotive industry [5]. PBS owns similar properties to some commodities widely used in the food industry. That is why PBS finds its main market in the food packaging industry [6,7,8,9]. Although it can fully disintegrate in compost soil, it is still an expensive polymer. For this reason, some research works have focused on manufacturing composite materials with natural fibers [10,11,12]. Thus, PBS has been used as a matrix for composites with hemp fiber [13], which contributes to lowering the overall cost of the developed material, making this a more environmentally friendly material. The use of wool waste in PBS composites has also been reported [14].

Spain is the second worldwide producer of almonds, just behind the USA. This industry generates a huge amount of waste, mainly shells. Although the use of almond shell waste to remove contaminants by adsorption has been reported [15,16,17], and they can be a biomass source for energy and fuels [18,19,20], one of the most attractive uses of almond shells is in the form of powder or flour (ASF), to be used in combination with several polymer matrices to give wood-like composite materials. Some investigations have focused on the partially biodegradable composites of poly(propylene) (PP) with almond shell flour [21,22], and fully biodegradable composites with poly(lactic acid) (PLA) matrices [23].

Almond shells are composed of approximately 38 wt % hemicellulose, 31 wt % cellulose, 28 wt % lignin, and 3 wt % other compounds [24]. This high content of cellulose, lignin, and hemicellulose gives a marked hydrophilic nature to the waste, which is opposite to the typical hydrophobic nature of most polymer matrices. This lack of (or very low) compatibility leads to stress concentration phenomena that, in turn, are responsible for a decrease in overall properties [25,26,27,28]. In particular, the mechanical properties of polymer-filled composites, which are directly related to material cohesion, are highly affected by the presence of hydrophilic particles embedded in a hydrophobic matrix. The elongation at break, as well as the impact strength are remarkably diminished [29,30]. To overcome this drawback, or at least to minimize its effects, several technical approaches are widely used such as the use of compatibilizers, surface treatment of particles that provide a more or less intense interaction between the polymer matrix and the embedded particles [31,32,33,34]. Plasticizers could act as internal lubricants, thus allowing chain mobility, which enhances processability and improves thermal stability and ductility. In the last decade, a new family of vegetable-oil-derived plasticizers has been proposed [35,36,37]. Among others, it is worth noting the increasing use of epoxidized oils such as linseed oil (ELO) [31,38], soybean oil (ESBO) [39], palm oil (EPO) [40], epoxidized castor oil (ECO) [41], epoxidized tung oil [42], and so on. Recently, the potential plasticization and compatibilization effects of maleinized oils such as linseed oil (MLO) [23,43], soybean oil (MSBO) [44], cotton seed (MCSO) [45,46], and hemp seed (MHSO) [47] have been reported.

Linseed oil (LO) is composed of about 9–11% saturated fatty acids (5–6% palmitic acid and 4–5% stearic acid) and around 75–90% unsaturated fatty acids (50–55% linolenic acid, 15–20% oleic acid, and 10–15% linoleic acid) [48]. This high unsaturated content allows chemical modification through different paths such as epoxidation, maleinization, hydroxylation, acrylation, etc. Maleinized linseed oil (MLO) has been proven to be a good plasticizer in PLA-based formulations, as reported by Ferri at al. [43]. In particular, they reported a dual effect of MLO on PLA blends with thermoplastic starch (TPS); on the one hand, a plasticization effect allows easy processing, and on the other hand, a compatibilizing effect through the reaction of maleic anhydride with hydroxyl groups in both PLA end chains and starch provides enhanced mechanical properties and thermal stability. It has also been proven to have its in poly(3-hydroxybutyrate) (PHB) formulations [36].

In a previous work with PBS composites and almond shell flour (ASF), maleinized linseed oil was revealed as the best compatibilizer compared to other families such as acrylates, anhydrides, and epoxidized oils [1]. This work is focused on the study of the effect of MLO loading on the final properties of PBS‒ASF composites with the aim of improving interface phenomena between the PBS matrix and the embedded ASF particles.

## 2. Materials and Methods 

### 2.1. Materials

A commercial grade of poly(butylene succinate) (PBS, Bionolle 1020MD, supplied by Showa Denko, Tokyo, Japan) was used as the base material for composites. This commercial grade possesses a melt flow index (MFI) comprised between 20 and 34 g/(10 min) and a density of 1.26 g cm^−3^.

Almond shell powder/flour (ASF) was purchased from Jesol Materias Primas (Valencia, Spain). The supplied powder was sieved in a vibrational sieve RP09 CISA® (Barcelona, Spain) to obtain a homogeneous particle size of 150 μm.

Maleinized linseed oil (MLO) was supplied by Vandeputte (Mouscron, Belgium) under the trade name of VEOMER LIN. It possesses a viscosity of 10 dPa, measured at 20 °C, and an acid value of 105–130 mg KOH g^−1^.

### 2.2. Manufacturing of PBS/ASF/MLO Composites

Initially, PBS and ASF were dried at 50 °C for 24 h to remove moisture since polyesters are highly sensitive to hydrolysis at high temperatures. MLO was heated to 40 °C to reduce its viscosity and enable good pre-mixing with PBS and ASF. Pre-mixing was carried out using ziploc bags with the appropriate compositions of each component (see Table 1). After this pre-mixing stage, the mixtures were compounded in a twin-screw co-rotating extruder from Dupra S.L. (Alicante, Spain) with a screw diameter of 25 mm and a length (L) to diameter (D) ratio (L/D) of 24. The screw speed was set to 40 rpm and the temperature profile was as follows: 120 °C, 125 °C, 130 °C, and 130 °C from the hopper to the die. After extrusion, the compounded materials were pelletized and dried at 50 °C for 24 h before further processing by injection molding in a Meteor 270/75 injection machine from Mateu & Solé (Barcelona, Spain). The temperature profile was set to 110 °C (hopper), 115 °C, 120 °C, and 125 °C (injection nozzle). 

### 2.3. Mechanical Characterization

Mechanical properties were obtained from standard tensile, hardness, and impact tests. Tensile tests were carried out in a universal test machine ELIB 50 from S.A.E. Ibertest (Madrid, Spain) following the guidelines of ISO 527-1:2012. A load cell of 5 kN was used and the crosshead speed was set to 10 mm min^−1^. Hardness was measured in a Shore D durometer model 676-D from J. Bot Instruments (Barcelona, Spain), as indicated by ISO 868:2003. With regard to impact tests, a Charpy’s pendulum with an energy of 1 J was used to obtain the impact energy on standard notched samples (“V” type notch with a radius of 0.25 mm), according to ISO 179-1:2010. At least five different samples were used for each test to ensure reproducible results and average values were calculated for each property. 

### 2.4. Morphological Characterization

Fractured samples from impact tests were observed by field emission scanning electron microscopy (FESEM) in a ZEISS ULTRA 55 microscope from Oxford Instruments (Abingdon, UK). All samples were previously subjected to a sputtering process with an Au‒Pd alloy to enhance electrical conductivity. The sputtering was carried out in a EMITECH SC7620 sputter coater from Quorum Technologies (Lewes, UK).

### 2.5. Thermal Characterization

The main thermal transitions were obtained by differential scanning calorimetry (DSC) using a Mettler-Toledo 821 calorimeter (Schwerzenbach, Switzerland). Samples with an average size of 5–7 mg were subjected to a dynamic thermal program consisting on three stages, a first heating from 25 °C to 200 °C, then a controlled cooling from 200 °C to −50 °C and, finally, a second heating process from −50 °C to 300 °C. The scanning rate was 10 °C min^−1^ for all three stages. All DSC runs were conducted in triplicate under a nitrogen atmosphere with a flow rate of 66 mL min^−1^. Standard sealed aluminum crucibles with a total volume of 40 µL were used. The degree of crystallinity was calculated by the following equation:

(1) $$X_{C}=\left[\frac{\Delta H_{m}}{\Delta H_{m}^{0}\;\times \;\left(1-w\right)}\right]\times 100,$$

where Δ*H_m_* stands for the measured melt enthalpy. Δ*H^0^_m_* (J g^−1^) represents the melt enthalpy of a theoretically fully crystalline PBS polymer, with a value of 110.3 J g^−1^ for PBS [49]. With regard to *w*, it represents the weight fraction of all added components (except for PBS), including both ASF and MLO. The thermal stability at elevated temperatures was followed by thermogravimetry (TGA) in a TGA/SDTA 851 from Mettler-Toledo. Standard alumina crucibles with a total volume of 70 mL were charged with approx. 6 mg and subsequently subjected to a dynamic heating program from 30 °C to 700 °C at a constant heating rate of 20 °C min^−1^ in air atmosphere.

### 2.6. Thermomechanical Characterization

Dynamic mechanical thermal analysis (DMTA) was conducted on a DMA1 analyzer from Mettler-Toledo. Single cantilever flexural tests were carried out at a frequency of 1 Hz at a heating rate of 2 °C min^−1^ and a maximum deflection of 10 μm. Rectangular samples with dimensions of 10 × 7 × 1 mm^3^ were subjected to a dynamic heating program from −50 °C to 80 °C.

Thermal stability of the developed composite materials was studied by thermomechanical analysis (TMA) in a Q400 TMA analyzer from TA Instruments (New Castle, DE, USA). A constant force of 0.02 N was applied to squared samples of 10 × 10 × 4 mm^3^ and subjected to a temperature sweep from −50 °C up to 80 °C at a constant heating rate of 2 °C min^−1^. The coefficient of linear thermal expansion (CLTE) was calculated both below and above the glass transition temperature (T_g_). All thermal runs were done in triplicate. 

## 3. Results

### 3.1. Effect of MLO Loading on Mechanical Properties of PBS/ASF Composites

Table 2 summarizes the main parameters obtained from mechanical characterization as a function of the MLO content.

As expected, the addition of ASF into the PBS matrix leads to an increase in stiffness together with a clear decrease in ductility. PBS possesses a σ_t_ of about 31.5 MPa; this is dramatically reduced by almost half (14.8 MPa) by filling the PBS matrix with 30 wt % ASF. As described previously, the highly hydrophilic nature of ASF does not allow interaction with the highly hydrophobic PBS matrix and this is responsible for poor load transfer from the filler to the matrix. In fact, the filler offers the typical stress concentration phenomenon due to a lack of interaction with the polymeric matrix. This lack of interaction contributes to poor material cohesion (small gaps between the particle filler and the surrounding matrix) and, therefore, the mechanical properties are worse than neat PBS. This stress concentration phenomenon is also evident when observing the extremely high decrease in ε_b_, which drops from 215.6% to 6.3% (a decrease of about 97%). In contrast, the material becomes stiffer, as shown by the E_t_ values. Neat PBS is a flexible polymer with a relatively low modulus of 417.4 MPa; this is almost doubled by the addition of 30 wt % ASF (787.9 MPa). This increase is evident as the modulus represents the ratio of the applied stress (σ) and the obtained elongation (ε) in the linear region. As has been shown in Table 2, the tensile strength decreases (by 50%) but the elongation at break decreases by 97%. Therefore, the σ/ε ratio gives higher values as the decrease in ε is much pronounced than that of σ. 

MLO has a dual effect on PBS/ASF composites. As the MLO load increases, both the tensile modulus and the tensile strength decrease due to a plasticizing effect, as reported by Ferri et al. [50] for PLA formulations containing 10–13 wt % MLO. Tensile strength and tensile modulus decrease in all formulations with increasing MLO content. In contrast, ductility is remarkably improved, as can be seen from the elongation at break values, which change from 6.3% (uncompatibilized PBS/ASF composite) to almost 26% for the PBS/ASF composite compatibilized with 4.5 wt % MLO. The elongation at break is not remarkably improved with higher MLO content. Quiles-Carrillo et al. [23] reported similar findings in PLA composites with ASF compatibilized with MLO. As an efficient plasticizer, MLO remarkably improved the ductility of the PLA/ASF composites. Elongation at break increased by 292% and 84% in relation to the uncompatibilized PLA/ASF and to the neat PLA, respectively. Ferri et al. [50] also reported a remarkable increase in elongation at break in PLA formulations with 10–13 wt % MLO. This increase in ductility is related to the lubricant effect of MLO, which increases chain mobility. The plasticizer increases the free volume and, subsequently, chain interactions decrease, leading to improved intermolecular mobility. Chieng et al. [35] also reported similar findings in PLA biocomposites plasticized with epoxidized vegetable oils (EVOs).

Regarding Shore D hardness, its evolution is identical to the tensile modulus. Neat PBS shows a Shore D value of 60.1. The addition of 30 wt % ASF leads to stiffer material with a Shore D value of 71.2. Then, as the MLO content increases, Shore D progressively decreased to values of 62.0 for the PBS/ASF composite with 10 wt % MLO.

The second effect of MLO can be inferred by observing the impact strength values in Table 2. Neat PBS is characterized by good energy absorption, which gives an impact strength of 16.5 kJ m^−2^. The addition of 30 wt % ASF causes a dramatic decrease in impact strength down to 1.8 kJ m^−2^. The impact strength is directly related to both ductility and resistance. As previously indicated, both the tensile strength and the elongation at break are remarkably reduced due to poor material cohesion, which does not allow stress transfer and, hence, there is low deformation with low applied stresses. Obviously, the ability to absorb energy is directly linked to the deformation capability, together with the need for high stress to promote deformation. Therefore, ASF addition leads to extremely low impact energy values. The addition of MLO provides a plasticizing effect, as indicated previously; in addition, reactions between MLO and both PBS and ASF could be expected as the absorbed energy increases to almost 4 kJ m^−2^ with 4.5 wt % MLO (which represents a percentage increase of more than 120% with respect to the uncompatibilized PBS/ASF composite). In fact, the maleic anhydride pendant group can react with hydroxyl groups in both PBS (end chains) and ASF particles (cellulose and hemicellulose). This effect has been previously reported by Ernzen et al. with polyamides and maleinized soybean oil [51], and Quiles-Carrillo et al. with polyesters [23,47] with maleinized linseed oil.

### 3.2. Effect of MLO Loading on Morphology of PBS/ASF Composites

**Figure 1:**
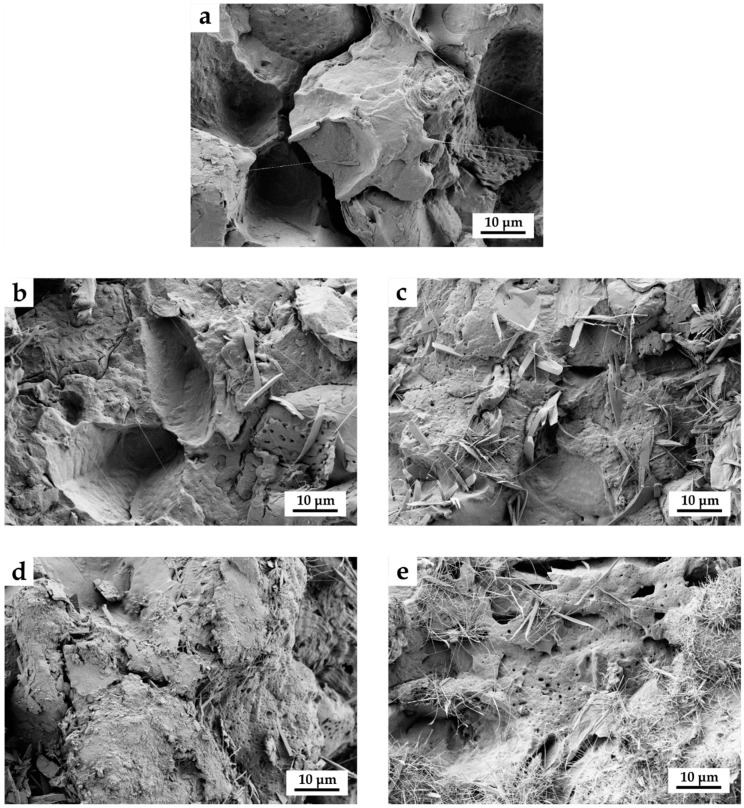
Field emission scanning electron microscopy (FESEM) images of the surface fracture of PBS/ASF composites, uncompatibilized and MLO-compatibilized taken at 1000×: (**a**) PBS + ASF; (**b**) PBS + ASF + 2.5MLO; (**c**) PBS + ASF + 4.5MLO; (**d**) PBS + ASF + 7.5MLO; and (**e**) PBS + ASF + 10MLO. Scale markers of 10 μm.

Figure 1 gathers FESEM images corresponding to PBS/ASF composites (uncompatibilized and MLO-compatibilized composites). As can be observed in Figure 1a, polymer‒particle adhesion is very poor. It is possible to find a small gap (1–3 µm) between the ASF particle and the surrounding PBS matrix. In addition, this lack of interaction is evident upon a detailed observation of the PBS fracture surface. PBS shows a porous pattern (1 µm) that is the reverse of that of ASF, which is identical to the typical surface of an almond shell at the macroscale [52]. The addition of 2.5 wt % MLO (Figure 1b) provides some interaction as the gap has been reduced. Nevertheless, some ASF particles have been pulled out without any interaction. Once again, it is possible to observe a negative copy of the almond shell microstructure on the PBS matrix in some areas. Compatibilization is more evident with higher MLO loads, as can be seen in Figure 1c, 1d, and 1e with 4.5, 7.5, and 10 wt % MLO, respectively. The typical PBS surface (negative copy of the ASF microstructure) after particle removal is not detected. The gap is almost inexistent and no signs of particle removal can be observed. All these issues indicate the good compatibilizing effect of MLO as it can react with both PBS and ASF.

### 3.3. Effect of MLO Loading on Thermal Properties of PBS/ASF Composites

**Figure 2:**
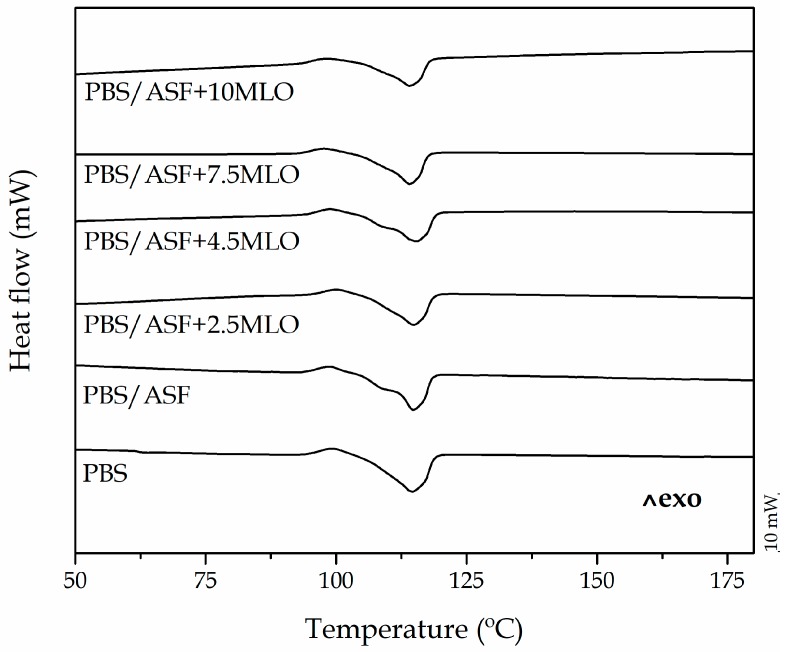
Differential scanning calorimetry (DSC) thermograms of neat PBS, uncompatibilized PBS/ASF composite, and PBS/ASF composites compatibilized with different MLO loading.

Table 3 and Figure 2 show a summary of the main thermal parameters obtained by DSC characterization of PBS/ASF composites. The melt peak temperature remains almost invariable after the addition of ASF and MLO and changes in a very narrow range from 113 °C to 116 °C [53]. A slight decrease in the melt peak temperature can be observed. This could be related to the nucleant effect a lignocellulosic filler exerts on semicrystalline polymers. Accordingly, the overall degree of crystallinity increases after the addition of ASF due to an interfacial phenomenon as the cellulose crystals act as nucleant points for PBS [54,55]. On the other hand, the addition of MLO provides a decrease in crystallinity down to values close to those of neat PBS. This effect is particularly noticeable for high MLO contents in the 7.5–10.0 wt % range. The plasticizing effect of MLO at higher concentrations provides increased chain mobility, which contributes to a slight decrease in the melt peak temperature down to 113 °C. It is worth noting the role of the melt temperature and crystallinity in the overall processing of polymers and their composites [56]. 

**Figure 3:**
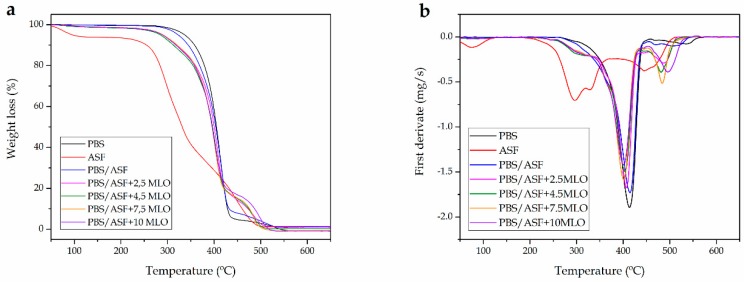
(**a**) Thermogravimetric (TGA) thermograms corresponding to raw ASF, neat PBS and PBS/ASF composites compatibilized with different MLO loading and (**b**) first derivative (DTG) curves.

With regard to thermal degradation at elevated temperatures, Figure 3a shows the TGA thermogram with the weight loss in function of increasing temperature. It has been reported that PBS decomposes by the following mechanisms, which cover typical random chain scission of aliphatic polyesters such as PLA [57], and specific chain scission. Shih et al. reported that the diffusion effect becomes important at elevated temperatures during degradation of PBS [58]. 

Addition of 30 wt % ASF into the PBS matrix provides a slight change in the TGA curves as the ASF degradation occurs in different stages. ASF shows the typical degradation profile of a lignocellulosic filler. At about 90–100 °C it is possible to observe a first weight loss that corresponds to the residual moisture contained in the ASF filler [59]. Over this temperature, ASF shows thermal stability up to 210–220 °C. Degradation of cellulose and hemicelluloses starts at about 220 °C and proceeds until 330–340 °C. Decomposition of cellulose and hemicellulose involves complex reactions (dehydration, decarboxylation, etc.) and the breakage of C–H, C–O, and C–C bonds. Lignin is more thermally stable and its degradation occurs in a broad temperature range starting at about 250 °C and ending at 450 °C in a progressive weight loss process. This higher thermal stability of lignin versus cellulose and hemicellulose is due to the presence of aromatic rings. Quiles-Carrillo et al. [60] showed similar degradation behavior in PLA/ASF composites. As has been reported by many authors, the degradation of lignocellulosic materials is a complex issue that mainly involves the degradation of hemicelluloses, cellulose, and lignin [61]. The components that most readily degrade due to a temperature rise are hemicelluloses, which decompose (depolymerize) in the temperature range 180–350 °C [62]. Crystalline areas of cellulose are more thermally stable and start to degrade at 275 °C; the decomposition takes place up to temperatures of 350–370 °C [63]. With regard to lignin, it shows a broader degradation range that starts at about 200 °C and lasts up to 700 °C, showing a typical shoulder at elevated temperatures in TGA thermograms [64]. The TGA curves corresponding to PBS/ASF composites show a slight decrease at the onset degradation temperature since the ASF filler is less thermally stable than PBS [11]. Sanchez-Jimenez at al. studied the thermal decomposition of cellulose and the typical degradation temperature range they reported overlaps with that of PBS [65]. It has been reported that the reactive extrusion of aliphatic polyesters such as PLA with styrene-epoxy acrylic oligomers can provide increased thermal stability due to the branching effect reactive extrusion provides [66]. As has been suggested in this study, MLO provides two overlapping processes: on one hand, a plasticizing effect and, on the other hand, a compatibilization effect due to the reaction of maleic anhydride with hydroxyl groups in PBS and ASF filler. Despite this, the thermal stability at moderate temperatures is not improved, as can be seen in Table 4, with a decreasing values of T_5%_ with increasing MLO. The same tendency can be observed for the maximum degradation rate temperature (T_max_) from 415.64. Despite this, it seems that the typical degradation peak of lignin at elevated temperatures is moved to higher temperatures with MLO addition, as can be observed in Figure 3b, which gathers the first derivative TGA curves for all PBS/ASF composites as well as raw ASF and neat PBS.

### 3.4. Effect of MLO Loading on Thermomechanical Properties of PBS/ASF Composites

**Figure 4:**
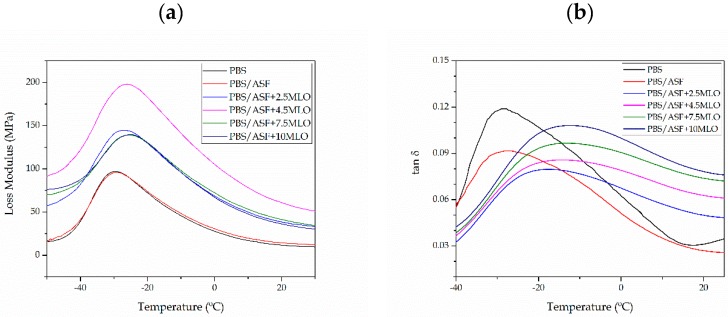
Plot evolution of (**a**) loss modulus (E″) and (**b**) damping factor (tan ∂) of neat PBS and PBS/ASF composites with different MLO compatibilizer content.

Figure 4 shows the evolution of the loss modulus (G″) and the damping factor (tan ∂). The glass transition temperature (T_g_) can be estimated (among other methods) by the peak temperature corresponding to the loss modulus (G″). It is important to remark that the T_g_ value could not be observed by DSC due to the low signal it gives. Neat PBS shows a T_g_ value of about −29 °C, which is in accordance with other T_g_ values reported in the literature [67]. The addition of 30 wt % ASF does not promote any change in the T_g_ value, thus indicating no interaction. In contrast to the expected behavior suggested by mechanical characterization, as the MLO increases the T_g_ also increases, in a slight way, but detectable by DMTA characterization. The compatibilizing effect of MLO by reaction with both PBS and ASF filler is evidenced by an increase in the T_g_ value since the reaction of MLO with both polymer matrix and particle filler restricts chain mobility and, hence, the T_g_ is increased. As reported by Quiles-Carrillo et al. [23] in a previous work, the effect of MLO is evident when comparing the FTIR spectra of uncompatibilized and compatibilized PLA/ASF composites. Comparison of these FTIR spectra indicate that new esters and carboxylic acids are obtained after reactive extrusion with PLA and ASF. They propose the formation of a cellulose-*g*-PLA by the reaction of the multiple anhydride groups in MLO with the hydroxyl groups in both PLA end chains and the lignocellulosic filler. Similar behavior could be expected for this system with a more flexible polymer (PBS) and the same lignocellulosic filler without previous ultraviolet (UV) surface treatment. PBS/ASF composites with 2.5 wt % MLO possess a T_g_ of −27 °C, which means a slight increase of 2 °C with respect to the PBS (or uncompatibilized PBS/ASF composite). As has been reported, MLO could potentially provide several overlapped effects such as plasticization, chain extension, branching, and even crosslinking. All these phenomena could overlap and, depending on the polymer, one mechanism could stand out over the others [50]. In this system, some plasticization is evident, as suggested by mechanical characterization, but, together with this phenomenon, compatibilization could occur, due to the reaction of maleic anhydride with PBS end chains and the lignocellulosic filler that restricts the Brownian motion of long-chain molecules [68]. On the other hand, it has also been reported that MLO could lead to some polymer crosslinking that could contribute to a slight increase in the T_g_. This increase in T_g_ is clear evidence of the compatibilization effect [69]. As the MLO content increases, a slight increase in T_g_ is observed as well. Therefore, for PBS/ASF composites compatibilized with 10 wt % MLO, the T_g_ reaches values of about −23.5 °C.

To obtain a complete characterization of the thermomechanical properties, the change in dimensions as a function of temperature was obtained by thermomechanical analysis (TMA). In particular, the coefficient of linear thermal expansion (CLTE) was calculated both below and above the glass transition temperature, as shown in Table 5. As PBS is extremely flexible, the comparison material has been the uncompatibilized PBS/ASF composite, which shows a CLTE of 59.3 µm m^−1^ °C^−1^. As previously indicated, the addition of MLO provides an increase in elongation due to a combined effect of plasticization and compatibilization through reaction. This increase in ductility is directly related to an increase in the CLTE. In fact, the CLTE shows a clear increasing trend with the MLO content, up to values of 84.8 µm m^−1^ °C^−1^ below the T_g_. This same tendency can be observed for the CLTE measured above the T_g_, as seen in Table 5.

## 4. Conclusions

Maleinized linseed oil (MLO) has been validated as a good compatibilizer in green composites of PBS and ASF, with the additional feature of being environmentally friendly. Uncompatibilized PBS/ASF composites show a dramatic decrease in both tensile strength and elongation at break due to the lack of polymer‒filler interaction. Accordingly, the impact strength of the uncompatibilized PBS/ASF composite is also reduced. MLO provides a combination of plasticization and compatibilization through the reaction of maleic anhydride pendant groups in the triglyceride structure with the hydroxyl groups contained in both PBS (end chains) and ASF (mainly cellulose and hemicelluloses). This leads to a noticeable increase in elongation at break as well as in the impact strength. MLO reaction with both PBS and ASF is also assessed by an increase in the T_g_ from −28 °C (neat PBS) up to −12 °C (PBS/ASF composite compatibilized with 10 wt % MLO). The compatibilization effect is also observed by scanning electron microscopy as the gap between the filler and the surrounding matrix almost disappears. Therefore, the herein-developed composites represent an interesting approach to reduce the overall cost of using PBS by using industrial waste coming from the almond industry, thus leading to highly environmentally friendly materials. These PBS/ASF composites can be successfully compatibilized with a vegetable-oil-derived additive, which contributes to the high environmental efficiency of these materials.

## Figures and Tables

**Table 1:** Composition and labelling of poly(butylene succinate) composites with almond shell flour (ASF) and different maleinized linseed oil (MLO) compatibilizing load.

Reference	PBS wt %	ASF wt %	MLO wt %
**PBS**	100.0	-	-
PBS + ASF	70.0	30.0	-
PBS + ASF + 2.5MLO	67.5	30.0	2.5
PBS + ASF + 4.5MLO	65.5	30.0	4.5
PBS + ASF + 7.5MLO	62.5	30.0	7.5
PBS + ASF + 10MLO	60.0	30.0	10.0

**Table 2:** Summary of mechanical properties of PBS/ASF composites with different MLO compatibilizer loading: tensile properties (tensile modulus—Et, tensile strength—σ_t_, and elongation at break—ε_b_), Shore D hardness and impact strength from Charpy test.

Reference	E_t_ (MPa)	σ_t_ (MPa)	ε_b_ (%)	Shore D Hardness	Impact Strength (kJ m^−2^)
PBS	417.4 ± 21.1	31.5 ± 0.9	215.6 ± 16.5	60.1 ± 0.5	16.5 ± 0.8
PBS + ASF	787.9 ± 55.8	14.8 ± 0.5	6.3 ± 0.9	71.2 ± 0.3	1.8 ± 0.3
PBS + ASF + 2.5MLO	779.8 ± 33.3	14.3 ± 0.5	17.4 ± 0.3	67.8 ± 0.9	2.5 ± 0.2
PBS + ASF + 4.5MLO	534.6 ± 51.3	13.8 ± 0.3	25.9 ± 1.0	67.2 ± 0.2	3.8 ± 0.5
PBS + ASF + 7.5MLO	423.4 ± 13.1	12.3 ± 0.1	26.1 ± 1.7	64.0 ± 0.4	3.9 ± 0.3
PBS + ASF + 10MLO	269.8 ± 30.7	11.7 ± 1.1	26.6 ± 1.2	62.0 ± 0.7	4.2 ± 0.2

**Table 3:** Main thermal parameters of neat PBS and PBS/AHF composites with different percentage of MLO obtained by differential canning calorimetry (DSC).

Code	Melt Enthalpy (J g^−1^)	Melt Peak Temperature, T_m_ (°C)	Xc (%)
PBS	68.9 ± 1.9	115.6 ± 2.3	60.9 ± 1.8
PBS/ASF	61.2 ± 1.7	115.1 ± 1.9	77.4 ± 1.6
PBS/ASF + 2.5MLO	58.6 ± 2.0	114.6 ± 1.7	79.6 ± 1.9
PBS/ASF + 4.5MLO	56.6 ± 2.1	115.2 ± 2.1	77.1 ± 2.0
PBS/ASF + 7.5MLO	47.3 ± 1.9	113.7 ± 1.7	66.9 ± 1.8
PBS/ASF + 10MLO	43.4 ± 1.5	113.9 ± 1.5	64.0 ± 1.4

**Table 4:** Summary of the main thermal parameters of the degradation process of PBS/ASF with different percentage of MLO.

Reference	* T_5%_ (°C)	** T_max_ (°C)	Residual Weight (%)
PBS	335.9 ± 1.8	415.64 ± 1.86	0.32 ± 0.12
PBS/ASF	329.6 ± 2.0	414.06 ± 1.98	1.23 ± 0.31
PBS/ASF + 2.5MLO	291.3 ± 2.1	407.18 ± 1.67	1.29 ± 0.32
PBS/ASF + 4.5MLO	288.7 ± 2.1	405.82 ± 2.08	0.68 ± 0.24
PBS/ASF + 7.5MLO	289.6 ± 1.4	400.47 ± 1.87	0.46 ± 0.32
PBS/ASF + 10MLO	289.3 ± 1.8	399.93 ± 1.57	0.42 ± 0.15

* T_5%_ represents the characteristic temperature for a mass loss of 5%. ** T_max_ represents the characteristic temperature corresponding to the maximum degradation rate.

**Table 5:** Variation of the coefficient of linear thermal expansion (CLTE) of neat PBS and PBS/ASF composites with different MLO compatibilizer content.

Reference	CLTE below T_g_ (µm m^−1^ °C^−1^)	CLTE above T_g_ (µm m^−1^ °C^−1^)
PBS/ASF	59.3 ± 1.6	128.1 ± 1.8
PBS/ASF + 2.5MLO	65.6 ± 1.6	132.1 ± 2.1
PBS/ASF + 4.5MLO	74.2 ± 2.1	138.7 ± 2.9
PBS/ASF + 7.5MLO	78.9 ± 2.3	142.9 ± 1.7
PBS/ASF + 10MLO	84.8 ± 1.9	144.1 ± 2.4
